# VEGF-B: a more balanced approach toward cardiac neovascularization?

**DOI:** 10.1002/emmm.201303730

**Published:** 2014-02-12

**Authors:** Christian Kupatt, Rabea Hinkel

**Affiliations:** 1 Medizinische Klinik I, Klinikum GroßhadernMunich, Germany; 2 DZHK (German Centre for Cardiovascular Research), Partner Site Munich Heart AllianceMunich, Germany

## Abstract

There is an urgent need for new pharmacologic approaches to combat the clinical consequences of ischemic cardiomyopathy. In this issue of *EMBO Molecular Medicine*, Kivelä *et al* show that transgenic expression of VEGF-B in the rat heart leads to expansion of the coronary arterial tree and an increase in functional coronary reserve, accompanied by a shift in myocardial metabolism from fatty acid to glucose utilization. See also: R Kivelä et al (March 2014)

Ischemic cardiomyopathy poses a great burden on patients and healthcare systems, in particular in industrialized countries. Although acute coronary care, reperfusion strategies of occluded coronary vessels and improvements in pharmacologic therapy are implemented in clinical treatment, mortality in patients with impaired heart function, for example failure to eject the expected amount of blood into the circulation, is still substantially increased. Pharmacologic innovations for heart failure have been scarce in the last decade. One promising molecular approach is gene therapy with recombinant adeno-associated vectors (rAAV) encoding the sarcoplasmatic reticulum calcium transporter SERCA. rAAV-SERCA, aimed at restoring appropriate ion handling in failing cardiomyocytes, is now being tested in clinical trials including CUPID1 (Jessup *et al*, 2011) and CUPID2 (ongoing).

An alternative approach has been to explore vascular gene therapy to improve perfusion of the chronic ischemic heart (Rissanen ' Yla-Herttuala, 2007). A therapeutic VEGF-A or FGF-vector was applied regionally (into a coronary vessel or directly into the myocardium), and alterations of heart perfusion and function were assessed. The results of these studies have not been clear-cut, and further translation of cardiovascular gene therapy was stalled.

Since then, the field has seen many improvements. Vectors now provide long-lasting and stable expression of transgenes. Magnetic resonance imaging allows for detailed assessment of cardiac perfusion and function. Moreover, novel vascular growth factors, lacking the known toxicity of VEGF-A or -C, have been studied. Furthermore, the need has been acknowledged to provide a more balanced vascular growth than that produced VEGF-A, including microvessel maturation as well as arteriogenesis (collateral growth) in addition to mere angiogenesis (Zacchigna *et al*, 2007; Kupatt *et al*, 2010).

“the need has been acknowledged to provide a more balanced vascular growth than that produced VEGF-A”

In this issue of *EMBO Molecular Medicine*, Kivelä *et al* (2014) now show that many of these criteria might be met by the vascular growth factor VEGF-B (Fig 1). The team led by Kari Alitalo report on the impact of transgenic or adeno-associated virus (AAV)-based VEGF-B overexpression on protection against acute myocardial infarction, signaling cascades, and metabolic sequelae. The authors' strategy has a solid foundation in the knowledge that VEGF-B overexpression produces broad angiogenic (Lahteenvuo *et al*, 2009), arteriogenic (Bry *et al*, 2010), and metabolic impacts (Hagberg *et al*, 2010).

**Figure 1:**
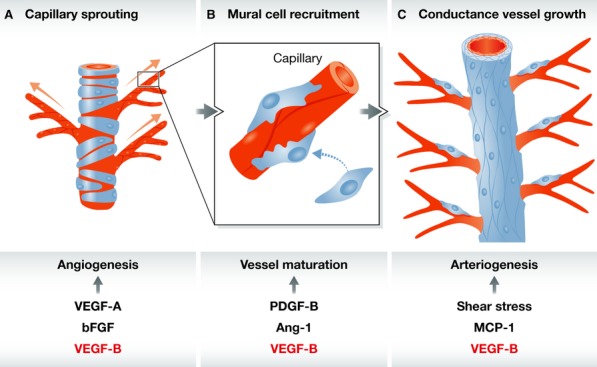
Angiogenesis (A) is followed by microvascular maturation (B), which triggers/enhances arteriogenesis (C). Kivelä *et al*. (2014) show that all three levels (balanced neovascularization) are induced by VEGF-B.

Kivelä *et al* (2014) find that a VEGF-B transgenic rat strain (expressing the 167 and 186 isoform) presented physiologic cardiac hypertrophy, without developing the cardiomyopathy reported in VEGF-B_167_ mice (Karpanen *et al*, 2008). Micro-CT investigation uncovered a larger network of arterioles and arteries in enlarged rat hearts, marking a notable species difference with respect to mouse hearts, which lacked the arterial growth. Consistently, an occlusion of a coronary artery in VEGF-B rats reduced infarct size at 1 and 4 weeks after the onset of myocardial infarction. The transgenic rat hearts clearly benefitted from the enhanced vascular network when a major vascular avenue was compromised. Accordingly, but perhaps worrying, human VEGF-B is less expressed in hearts suffering from cardiomyopathies.

By unraveling the underlying signaling cascades, the authors show a profound effect of VEGF-B on VEGF receptor-2 (VEGFR-2), extending the previously known effect on VEGFR1. Downstream kinase signaling includes Erk1/2, AKT, and the mTORC1 complex, which is expected for a VEGFR-2-ligand. Interestingly, although nitric oxide is capable of inducing or facilitating vascular growth, hypertrophy, and cardioprotection against ischemic events (Kupatt *et al*, 2007; Jaba *et al*, 2013), it does not seem to mediate VEGF-B-induced hypertrophy.

The mTORC1 complex is a well-known metabolic hub in the heart. Gain- and loss-of-function rat strains show opposite effects on fatty acid catabolism genes: VEGF-B transgenic rats display a decrease in Pdk4 as well as an increased FatP4 activity in cardiomyocytes, the exact opposite to VEGF-B-knockout rats. Still, fatty acid uptake does not appear to be altered in transgenic or AAV-transduced VEGF-B-overexpressing hearts, in contrast to the previous reports in mice (Hagberg *et al*, 2010). Mitochondrial activity at complex I, however, was maintained at higher rates in VEGF-B transgenic rats, potentially implying a role of mitochondrial function in the improved ischemic resistance of VEGF-B-exposed tissue. Future work will decipher the hierarchy of events in structural and metabolic alterations after VEGF-B exposure.

In summary, the authors have shown that increasing VEGF-B, which is surprisingly unessential for heart development, by genetic means or AAV transduction induces physiologic hypertrophy of the heart and cardioprotection after myocardial infarction. VEGF-B appears to rely on VEGFR-1 and -2 to provide a balanced growth of capillaries, microarterioles, and conductance vessels. Since balanced vascular growth, that is, providing mature micro- and macrovessels, appears essential for the treatment of patients with chronic ischemic diseases, this feature may constitute a distinct progress in translating therapeutic neovascularization into patient care. It remains to be seen, however, whether the findings on VEGF-B in the rat can be repeated in other species, or whether the less beneficial mouse phenotype prevails.
